# The prevalence of anemia among type 2 diabetes mellitus with respect to renal function at a tertiary care hospital: A case-control study

**DOI:** 10.6026/973206300220353

**Published:** 2026-01-31

**Authors:** Yugantar Wakde, Prem Chand Dhruw, Pallavi Kumari

**Affiliations:** 1 Department of Biochemistry, Shri Rawatpura Sarkar Institute of Medical Sciences and Research, Naya Raipur, Chhattisgarh, India; 2 Department of Pathology, Shri Rawatpura Sarkar Institute of Medical Sciences and Research, Naya Raipur, Chhattisgarh, India

**Keywords:** Anemia, diabetes mellitus, HbA1c, hemoglobin, renal function

## Abstract

The prevalence of anemia in type 2 diabetes mellitus (T2DM) patients, particularly associated with renal dysfunction, remains inadequately studied. Hence, a total of 220 subjects were recruited, with 110 T2DM patients and 110 non-diabetic controls. The Hb levels of T2DM patients were 9.2 g/dl, compared to 13.5 g/dl in controls. Urea levels for T2DM patients were 33.6 mg/dl, while controls had 18.2 mg/dl. Creatinine levels were 1.78 mg/dl in T2DM patients, and 0.81 mg/dl in controls. Thus, we show the prevalence of anemia among T2DM patients attending our hospital. Results indicate a higher prevalence of anemia in T2DM patients, predominantly associated with renal dysfunction.

## Background:

Diabetes Mellitus (DM) are a group of metabolic disorders which has the phenotype of hyperglycemia. DM is a global endemic with rapidly increasing prevalence in both developing and developed countries [1]. In addition to hyperglycemia, patients with diabetes may develop macroangiopathy, microangiopathy and neuropathy. Diabetic nephropathy (DN) is one of the most common microvascular complications of diabetes mellitus, occurring in approximately 40-50% of patients with diabetes [2]. WHO has declared India as "Diabetic Capital of the world". The prevalence of both type 1 and type 2 DM are increased, type 2 DM is expected to rise more rapidly in future because of increased obesity and reduced activity levels. The chronic complications of DM affect many organ systems and are responsible for the majority of morbidity and mortality associated with the disease [3]. Glycated hemoglobin (HbA1c) is routinely used as a diagnostic tool for measuring long term glycemic control. In accordance with its function as an indicator for the mean blood glucose level, HbA1c predicts the risk for the development of diabetic complication in diabetes patients. The chronic hyperglycemia of diabetes is associated with damage and failure of various organs, especially the eyes, kidneys, nerves, heart, and vascular system [4]. Diabetes is the major cause of end-stage renal disease and diabetic nephropathy which are also called as diabetic kidney disease. Urea and Creatinine are the parameters to diagnose functioning of the kidneys. Changes in serum Creatinine concentration more reliably reflect changes in GFR than do changes in serum Urea concentration [5]. DN is one of the leading causes of end-stage renal disease, posing a significant economic and medical burden. DN pathogenesis is primarily related to the accumulation of glycation products, insulin resistance, oxidative stress, inflammatory response and other mechanisms. Additionally, impaired endothelial cell function is an important factor in DN progression [6]. Studies have reported that the deformability of red blood cells may be one of the mechanisms underlying diabetic microvascular complications, which is critical in the progression of DN and diabetic foot. Progressive impairment of red blood cell deformability is associated with the loss of renal function in patients with diabetes, and changes in hemoglobin (Hb) levels are essential for red blood cell deformability [7]. Notably, the Diabetes Control and Complications Trial showed a significant relationship between reduction in glycosylated hemoglobin (HbA1c) levels and the risk of microvascular complications including chronic kidney disease [8]. Therefore, it is of interest to analyze the correlation between Hb level and renal functions among Type 2-DM (T2DM).

## Materials and Methods: 

This cross-sectional study was conducted in the Department of Biochemistry and Department of Pathology, SRIMSR, Naya Raipur, Chhattisgarh, India. The study was conducted in between January 2025 to June 2025. A total of 220 subjects were recruited into the study using simple random sampling method. Out of 220, T2DM patients were 110 and non-diabetic subjects were 110.Inclusion criteria Diabetic and non-diabetic subjects willing to participate in the study, age of ≥18 years, both male and females were included. T2DM was diagnosed based on the American Diabetes Association criteria. Exclusion criteria Patients refused to participate in the study, patients with immunosuppressive therapy, autoimmune diseases, cardiovascular diseases, diabetes other than T2DM, liver diseases, renal diseases, thyroid diseases, gestational diabetics, epilepsy, hypertensive encephalopathy, malignancy conditions, and pregnant women were excluded from the study. Under aseptic conditions, 5 mL of venous blood samples were collected from all study subjects, visiting to Department of General Medicine. Blood samples are divided into two tubes: 2 mL had drawn into ethylene diamine tetra acetic acid (EDTA) tubes to measure the hematological parameter; complete blood count (CBC), and biochemical parameter; HbA1c and 3 mL into a plain tube with no anticoagulant to measure the biochemical parameters. The collected blood samples were allowed to stand for 1 hour and centrifuged at 3000 rpm for 10 minutes to separate serum sample. The obtained serum sample was used for the estimation of urea (urease), creatinine (Jaffe's) were estimated using Mindray BS-240 Biochemistry fully auto-analyzer. EDTA samples were used for the measurement of HbA1c using Agappe Mispa-i2 analyzer and CBC using Nihon Kohden Hematology analyzer. Detailed physical and clinical examination was done for all the study subjects. Body mass index (BMI) was also calculated. Statistical analysis was performed using SPSS version 20. Mean and standard deviation (SD) were calculated for continuous variables. Independent sample t-tests applied to compare means between T2DM and non-diabetic control subjects. A p-value of <0.05 was considered statistically significant throughout the study.

## Results:

Table 1 shows demographic details of T2DM and non-diabetic subjects. The mean age of T2DM is 53.2 and non-diabetic is 48.9 with SD 10.1 and 11.8 respectively. Out of total 110 patients, 52 are male while 58 are female that is 47.2% and 52.7% respectively. The BMI of T2DM patients are 26.4 with SD 3.1 and non-diabetic subjects are 21.7 with SD1.9. BMI values are statistically significant. Table 2 shows comparison of CBC parameters among all subjects. The Hb levels of T2DM are 9.2 g/dl and for control subjects are 13.5 g/dl with SD 1.8 and 2.4 respectively. The RBC levels of T2DM are 4.2 and for control subjects are 5.8 with SD 0.9 and 0.7 respectively. The PCV % of T2DM is 32.7 and for control subjects are 45.6 with SD 5.4 and 5.1 respectively. The MCV levels of T2DM are 72.6 and for control subjects are 86.3. The MCH levels of T2DM are 21.6 and for control subjects are 30.9 with SD 0.33 and 0.29 respectively. All the values are statistically significant. Table 3 reflects comparison of renal parameters among study subjects. The urea levels of T2DM are 33.6 mg/dl and for control subjects are 18.2 mg/dl with SD 8.2 and 6.5 respectively. The Creatinine levels of T2DM are 1.78 mg/dl and for control subjects are 0.81mg/dl with SD 0.62 and 0.17 respectively. All the values are statistically significant.

## Discussion:

Our study aimed to assess the prevalence and severity of anemia among T2DM patients attending in our hospital. Our study found a gender difference in the prevalence of anemia among patients with diabetes, with a significantly higher prevalence among females compared to males. The higher prevalence of anemia in diabetic females compared to diabetic males can be attributed to several factors: increased risk of iron deficiency due to menstrual blood loss and pregnancy, dietary patterns that may lead to inadequate intake of iron and other essential nutrients, and a higher prevalence of chronic kidney disease, a common complication of diabetes and a significant risk factor for anemia. In our study, severe anemia was significantly more prevalent among patients with uncontrolled T2DM. This finding correlate with many studies conducted, such results from studies suggest the negative impact of poor diabetic control on anemia [9]. It has been postulated that anemia associated with alterations in hemoglobin, delayed turnover and increased lifespan of red blood cells (RBCs), this factors contribute towards increase in glycation of hemoglobin [10]. Glycated hemoglobin (HbA1c) is a traditional tool to assess glycemic control over the last three months, among diabetic individuals. Anemia, hemoglobinopathies, acute and chronic blood loss, pregnancy, and uremia can also affect HbA1c levels [11].

Our study suggests that HbA1c has a positive correlation with CBC parameters like hemoglobin, MCV, MCH and MCHC in non-diabetic subjects with anemia, indicating an inverse relationship with anemia. On further evaluation, HbA1c was also found to be significantly reduced in patient with anemia compared to those with control subjects. Decreased hemoglobin, MCV, MCH and MCHC levels are indicative of anemia. Based on these findings, we suggest that HbA1c decreases with severity of anemia. There is limited literature available showing the relationship of HbA1c with anemia severity among non-diabetic subjects. In our study we found that the correlation of anemia with renal parameters among T2DM compared with non-diabetic subjects. The some study reflect the same but the mechanisms for this correlation are unknown [12]. The significant correlation between lower hemoglobin levels in T2D patients with increased age and worse kidney function. Anemia's are mostly caused by a nutritional deficiency of either iron or vitamins. Renal- related anemia is also sometime caused by nutritional deficiencies in addition to a functional erythropoietin deficiency or inflammation [13]. The association between anemia and impaired kidney function has been highlighted by multiple studies, with lower hemoglobin encountered in patients of anemia being considered a strong predictor of prognosis in patients with T2DM and renal failure [14]. The limitation of our study, which does not allow a time dependent analysis of the interaction between anemia, renal dysfunction and complications of diabetes.

## Conclusion:

The prevalence of anemia in patients with T2DM is high and it is associated with renal dysfunction. The presence of anemia in T2DM patients is significantly correlated with impaired kidney function. This study found a negative correlation of HbA1c with CBC parameters. Patients with uncontrolled diabetes had a high risk of developing severe anemia and renal failure. Hence, it is advised for the routine anemia and or CBC screening of patients with diabetes to facilitate early detection and effective management, to improve overall patient health. However, more data is needed to uderstand the mechanisms between T2DM and anemia.

## Figures and Tables

**Table 1:** Demographic details of the subjects

	**T2DM(n=110)**	**Non-diabetic (n=110)**	**P-value**
Age(years)	53.2±10.1	48.9±11.8	<0.05
Male (%)	52(47.2%)	61(55.4%)	---
Female (%)	58(52.7%)	49(44.5%)	---
BMI(kg/m^2^)	26.4±3.1	21.7±1.9	<0.05

**Table 2:** Comparison of CBC parameters

	**T2DM**	**Non-diabetic**	**P-value**
Hb(g/dl)	9.2±1.8	13.5±2.4	<0.05
RBC(10^6^/uL)	4.2±0.9	5.8±0.7	<0.05
PCV(%)	32.7±5.4	45.6±5.1	<0.05
MCV(fl)	72.6±9.5	86.3±8.7	<0.05
MCH(pg)	21.6±0.33	30.9±0.29	<0.05
MCHC(g/dl)	29.7±1.56	33.2±2.81	<0.05

**Table 3:** Comparison of renal parameters

	**T2DM**	**Non-diabetic**	**P-value**
Urea(mg/dl)	33.6±8.2	18.2±6.5	<0.05
Creatinine(mg/dl)	1.78±0.62	0.81±0.17	<0.05

## References

[1] BR P (2025). Oral Sphere J. Dent. Health Sci..

[2] Zhang X (2024). J Am Heart Assoc..

[3] Shah A (2013). J Diabetes Metab Disord..

[4] Sherwani SI (2016). Biomark Insights..

[5] Dabla PK (2010). World J Diabetes..

[6] Liu X (2025). Acta Diabetol..

[7] Tao L (2025). BMC Endocr Disord..

[8] https://www.ncbi.nlm.nih.gov/books/NBK549816/.

[9] AlDallal SM (2018). J Hematol..

[10] Janice D (2022). Int J Biochem Mol Biol..

[11] Tao X (2023). Sci Rep..

[12] Abass AE (2017). Clin Pract..

[13] Feteh VF (2016). BMC Nephrol..

[14] Song HY (2021). World J Diabetes..

